# Toxic fluoride gas emissions from lithium-ion battery fires

**DOI:** 10.1038/s41598-017-09784-z

**Published:** 2017-08-30

**Authors:** Fredrik Larsson, Petra Andersson, Per Blomqvist, Bengt-Erik Mellander

**Affiliations:** 1 Department of Physics, Chalmers University of Technology, Kemivagen 9, SE-41296 Gothenburg, Sweden; 2 Safety and Transport, RISE Research Institutes of Sweden, Brinellgatan 4, SE-50115 Boras, Sweden

## Abstract

Lithium-ion battery fires generate intense heat and considerable amounts of gas and smoke. Although the emission of toxic gases can be a larger threat than the heat, the knowledge of such emissions is limited. This paper presents quantitative measurements of heat release and fluoride gas emissions during battery fires for seven different types of commercial lithium-ion batteries. The results have been validated using two independent measurement techniques and show that large amounts of hydrogen fluoride (HF) may be generated, ranging between 20 and 200 mg/Wh of nominal battery energy capacity. In addition, 15–22 mg/Wh of another potentially toxic gas, phosphoryl fluoride (POF_3_), was measured in some of the fire tests. Gas emissions when using water mist as extinguishing agent were also investigated. Fluoride gas emission can pose a serious toxic threat and the results are crucial findings for risk assessment and management, especially for large Li-ion battery packs.

## Introduction

Lithium-ion batteries are a technical and a commercial success enabling a number of applications from cellular phones to electric vehicles and large scale electrical energy storage plants. The occasional occurrences of battery fires have, however, caused some concern especially regarding the risk for spontaneous fires and the intense heat generated by such fires^1–5^. While the fire itself and the heat it generates may be a serious threat in many situations, the risks associated with gas and smoke emissions from malfunctioning lithium-ion batteries may in some circumstances be a larger threat, especially in confined environments where people are present, such as in an aircraft, a submarine, a mine shaft, a spacecraft or in a home equipped with a battery energy storage system. The gas emissions has however only been studied to a very limited extent.

An irreversible thermal event in a lithium-ion battery can be initiated in several ways, by spontaneous internal or external short-circuit, overcharging, external heating or fire, mechanical abuse etc. This may result in a thermal runaway caused by the exothermal reactions in the battery^6–10^, eventually resulting in a fire and/or explosion. The consequences of such an event in a large Li-ion battery pack can be severe due to the risk for failure propagation^11–13^. The electrolyte in a lithium-ion battery is flammable and generally contains lithium hexafluorophosphate (LiPF_6_) or other Li-salts containing fluorine. In the event of overheating the electrolyte will evaporate and eventually be vented out from the battery cells. The gases may or may not be ignited immediately. In case the emitted gas is not immediately ignited the risk for a gas explosion at a later stage may be imminent. Li-ion batteries release a various number of toxic substances^14–16^ as well as e.g. CO (an asphyxiant gas) and CO_2_ (induces anoxia) during heating and fire. At elevated temperature the fluorine content of the electrolyte and, to some extent, other parts of the battery such as the polyvinylidene fluoride (PVdF) binder in the electrodes, may form gases such as hydrogen fluoride HF, phosphorus pentafluoride (PF_5_) and phosphoryl fluoride (POF_3_). Compounds containing fluorine can also be present as e.g. flame retardants in electrolyte and/or separator^17^, in additives and in the electrode materials, e.g. fluorophosphates^18,19^, adding additional sources of fluorine.

The decomposition of LiPF_6_ is promoted by the presence of water/humidity according to the following reactions^20,21^;

1 $${{\rm{LiPF}}}_{6}\to {\text{LiF}+\text{PF}}_{5}$$

2 $${{\rm{PF}}}_{5}{+{\rm{H}}}_{2}{\rm{O}}\to {{\rm{POF}}}_{3}\,+\,\text{2HF}$$

3 $${{\rm{LiPF}}}_{6}{+{\rm{H}}}_{2}{\rm{O}}\to {\text{LiF}+\text{POF}}_{3}\,+\,\text{2HF}$$

Of these PF_5_ is rather short lived. The toxicity of HF and the derivate hydrofluoric acid is well known^22–24^ while there is no toxicity data available for POF_3_, which is a reactive intermediate^25^ that will either react with other organic materials or with water finally generating HF. Judging from its chlorine analogy POCl_3_/HCl^24^, POF_3_ may even be more toxic than HF. The decomposition of fluorine containing compounds is complex and many other toxic fluoride gases might also be emitted in these situations, however, this study focuses on analysis of HF and POF_3_.

Although a number of qualitative and semi-quantitative attempts have been made in order to measure HF from Li-ion batteries under abuse conditions, most studies do not report time dependent rates or total amounts of HF and other fluorine containing gases for different battery types, battery chemistries and state-of-charge (SOC). In some measurements reported, HF has been found, within limited SOC-variations, during the abuse of Li-ion battery cells^15,16,26^, as well as detected during the abuse of battery packs^27^. However, time-resolved quantitative HF gas emission measurements from complete Li-ion battery cells undergoing an abusive situation have until now only been studied to a limited extend; for a few SOC-values, including larger commercial cells^28,29^, a smaller-size commercial cell^30^ and a research cell (i.e. non-commercial cell)^31^. Time-resolved quantitative HF measurements on the gas release from complete electric vehicles including their Li-ion battery packs during an external fire have also been performed^32^. Other types of gas emissions from Li-ion cells during abuse have been the subject of a somewhat larger number of investigations^33–41^. Since the electrolyte typically is the primary source of fluorine, measurements of fluorine emissions from battery type electrolytes have been studied. For example, fire or external heating abuse tests have been performed on electrolytes^42–46^ and the quantitative amounts of HF and POF_3_ have been measured in some cases^45,46^. Other studies of electrolytes exposed to moderate temperatures, 50–85 °C, show the generation of various fluorine compounds^20,21,47–49^ and some studies include both electrolyte and electrode material^50,51,52^.

Our quantitative study of the emission gases from Li-ion battery fires covers a wide range of battery types. We found that commercial lithium-ion batteries can emit considerable amounts of HF during a fire and that the emission rates vary for different types of batteries and SOC levels. POF_3_, on the other hand, was found only in one of the cell types and only at 0% SOC. The use of water mist as an extinguishing agent may promote the formation of unwanted gases as in eqs (2)–(3) and our limited measurements show an increase of HF production rate during the application of water mist, however, no significant difference in the total amount of HF formed with or without the use of water mist.

### Lithium-ion battery fire tests

The experiments were performed using an external propane burner for the purpose of heating and igniting the battery cells as described in the Methods section. Seven different types of batteries, type A-G, were investigated, from seven manufacturers and with different capacity, packaging type, design and cell chemistry, as specified in Table 1. Type A had a lithium cobalt oxide (LCO) cathode and carbon anode, types B to E had lithium-iron phosphate (LFP) cathode and carbon anode, type F had nickel cobalt aluminum oxide (NCA) and lithium aluminum titanium phosphate (LATP) electrodes while type G was a laptop battery pack with unspecified battery chemistry. All electrolytes contained LiPF_6_. Most of the cells were tested for different SOC levels, from fully charged, 100% SOC, to fully discharged, 0% SOC. The study included large-sized automotive-classed cells, i.e. series production cells of high industry quality, with long life time etc.

**Table 1:** Details of the tested Li-ion battery cells.

Battery	Numbers of batteries per test	Type	Nominal capacity per battery (Ah)	Nominal voltage per battery (V)	Cell packaging
A	5–10	LCO (LiCoO_2_)	6.8	3.75	Prismatic hard Al-can
B	2	LFP (LiFePO_4_)	20	3.2	Pouch
C	5	LFP (LiFePO_4_)	7	3.2	Pouch
D	9	LFP (LiFePO_4_)	3.2	3.2	Cylindrical
E	5	LFP (LiFePO_4_)	8	3.3	Cylindrical
F	2	NCA-LATP (LiNiCoAlO_2_-LiAlTiPO_4_)	30	2.3	Pouch
G	2	Laptop pack*	5.6	11.1	Cylindrical

^*^Each laptop battery pack has 6 cells of type 18650; arranged 2 in parallel and 3 in series.

**Figure 1:**
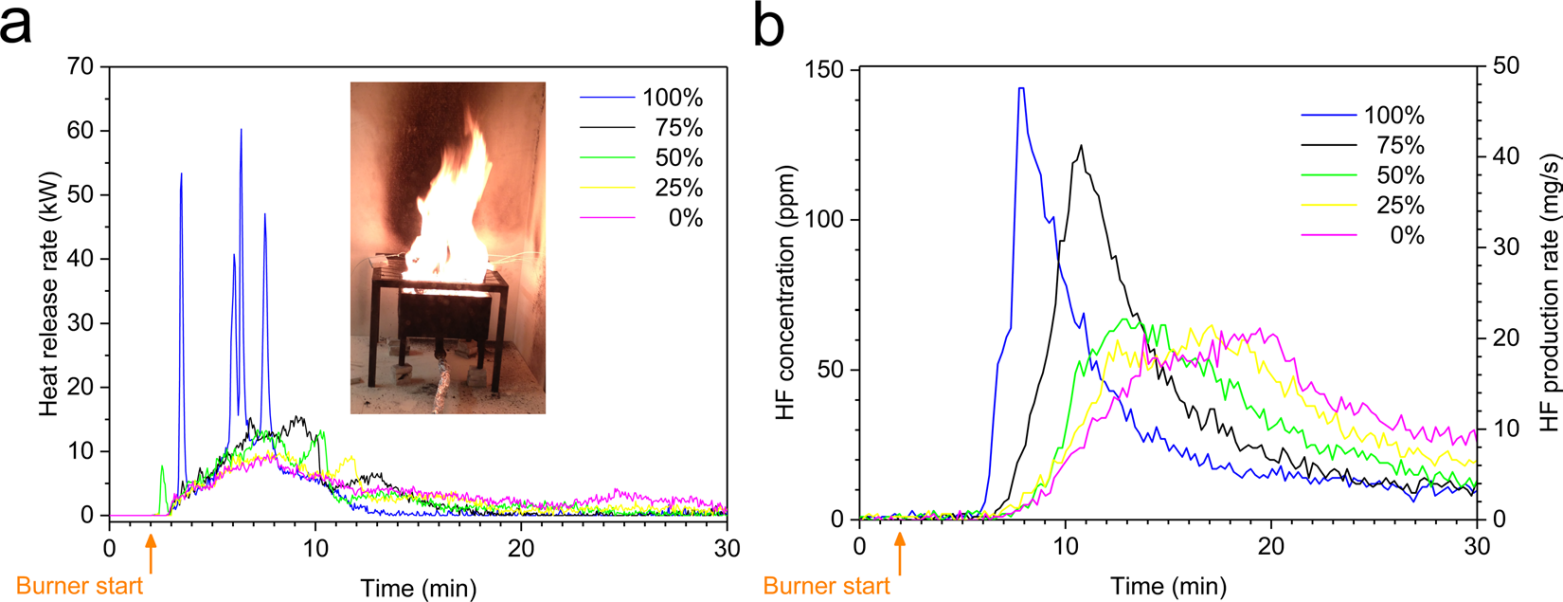
Results for type B cells, for 0–100% SOC with intermediate SOC-steps of 25%, exposed to an external propane fire; (**a**) showing the heat release rate (burner HRR contribution is subtracted), the inset photo shows burning battery cells during the test; (**b**) showing the HF release both as the measured concentrations as well as the calculated HF production rates. The HF production rates are calculated from the measured HF concentration by the Ideal gas law taking into account the ventilation flow, see Methods. The starting time of the heating process is marked on the time axis.

The heat release rate (HRR) and the emitted HF for B-type cells with different SOC values are shown in Fig. 1. Only the 100% SOC cells show several distinct peaks, corresponding to intense flares, when the cells vented and the emitted gas burn, for all other cells the heat release as a function of time is more smooth. These behaviors are reproducible also for the other tested cell types, e.g., only the 100% SOC cells show the more violent heat release peaks with intense flares.

**Figure 2:**
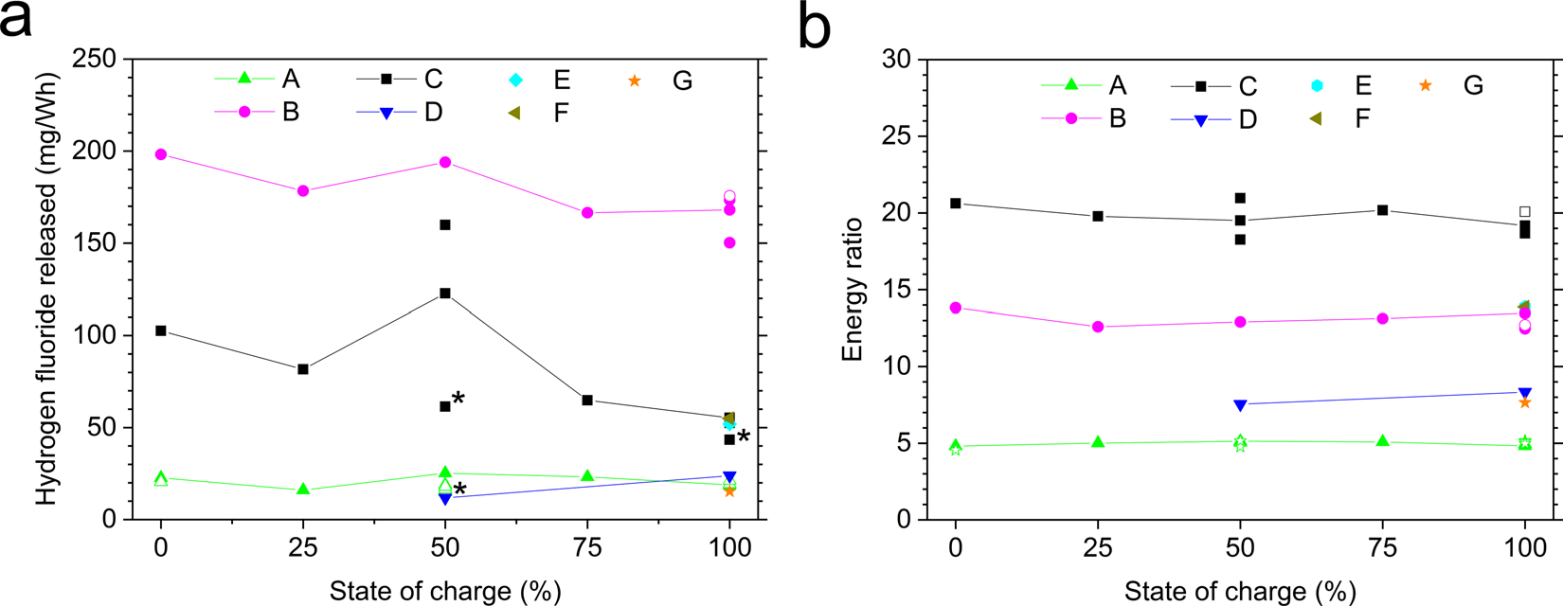
Total amount of HF measured by FTIR, normalized to nominal electrical energy capacity (**a**) and the energy ratio (**b**), for seven types of Li-ion battery cells and with various state of charge levels. Non-filled symbols indicate a repetition variant, e.g. applying water mist. The lines are intended as a guide for the eye. The energy ratio is a dimensionless value calculated by taking the total heat release from the battery fire divided by the nominal electrical energy capacity. Note that for 100% SOC the values are overlapping for type C, E and F as well as for type A, D and G in (**a**) and type B, E and F in (**b**). *Low value for type C at 50% and 100% SOC and type D at 50% SOC due to that a pre HF-saturation was not applied, therefore a part of the HF release was likely to be saturated in the gas sampling system, see Methods.

The measurements of the gas emissions during the fire tests show that the production of HF is correlated to the increase in HRR although somewhat delayed. From Fig. 1b it is evident that the higher SOC value, the higher values for the peak HF release rate. The total amount of HF varies considerably for the different battery types, see Fig. 2a. The amount of HF produced, expressed in mg/Wh, where Wh is the nominal battery energy capacity, is approximately 10 times higher for the cell with the highest values compared to the cells with the lowest values. The different relative amount of electrolyte and filler materials in the cells could be the simple explanation of this variation but information on those amounts are difficult to access for commercial batteries. The highest HF values are found for the pouch cells, a possible explanation would be that hard prismatic and cylindrical cells can build a higher pressure before bursting, rapidly releasing a high amount of gases/vapors from the electrolyte. Due to the high velocity of the release and thus the short reaction time, combustion reactions might be incomplete and less reaction products might be produced. In the test involving type G the cylindrical cells were layered horizontally, thus having a different venting direction and possibly increased wall losses, which combined with a very energetic response, might suggest why HF was detected only from the filter analysis and not detected by FTIR-analysis. The tested pouch cells of type B and C burned for longer time and with less intensity. The pouch cell of type F, however, burned faster, possibly due to its different electrode materials. The SOC influence on the HF release was less significant and the trend in Fig. 2a shows higher HF values for 0% than for 100% SOC, however with clear peaks at 50% SOC. Although these results are reproducible, they are difficult to explain. In other studies^30,31^, significantly narrower in test scope, involving smaller-sized cells and using a somewhat different abuse method, it was found that the total amount of HF measured by real-time FTIR was higher for decreasing SOC (tests conducted at 100%, 50% and 0% SOC).

The HRR curve is used to calculate the total heat release (THR) which corresponds to the energy released from the burning battery. THR is obtained by integrating the measured HRR (with the burner contribution subtracted) over the complete test time. Fig. 2b shows the energy ratio, that is how much energy is produced by the burning battery, compared to the amount of nominal electrical energy capacity a fully charged battery can deliver to an external circuit. The energy ratio is therefore a comparison between the chemical and the electrical energy of the Li-ion battery cell. The energy ratio varies considerably for the different cell types but is approximately constant for each cell, independent of SOC level. There are some similarities in Fig. 2a and b for the pouch cells, type B and C, which give the highest values in both cases, although in reverse order. This might indicate a higher amount of combustibles, e.g. electrolyte, in these cells compared to the other cells. It is also interesting to see that the energy ratio varies significantly between the tested cells, ranging from 5 to 21. This is important knowledge for fire protection and fire fighting. The energy ratio thus refers to a nominal fully charged battery while in normal use only a part of the SOC-window is used, for example half (50%) of the SOC-window (corresponding to cycling the battery between e.g. 30% and 80% SOC). If instead, the total heat release divided by the used electric battery capacity in the specific application is considered, higher energy ratio values are obtained. A summary of the results is shown in Table 2.

The measured heat release from an overheated battery may include several aspects, e.g. the battery temperature increase and the combustion of released gases. Variations due to the type of battery cell, the initiation method, e.g. if the test is done as an external fire test, an external heating or an overcharge test, and the test method, e.g. access to ambient oxygen (inert, under-ventilated or well-ventilated fire), and the presence of an external igniter, can greatly affect the amount of measured heat release. Energy release from a internal cell event in a confined environment can, for example, be lower than the energy release from the same cell in case of external fire. Thus energy ratios published using other methods and other types of Li-ion cells can be significantly different^7,52,53^.

For all tested battert types and selected SOC-levels, POF_3_ could only be measured quantitatively for type A battery cells at 0% SOC. Repeated measurements confirmed the presence of POF_3_ only for type A and only for 0% SOC. No POF_3_ could thus be detected in any of the other tests. POF_3_ is an intermediate compound and the local combustion conditions in every test, will influence the amounts of POF_3_ generated. This shows the importance of investigating many different set-ups when evaluating emitted gases.

**Figure 3:**
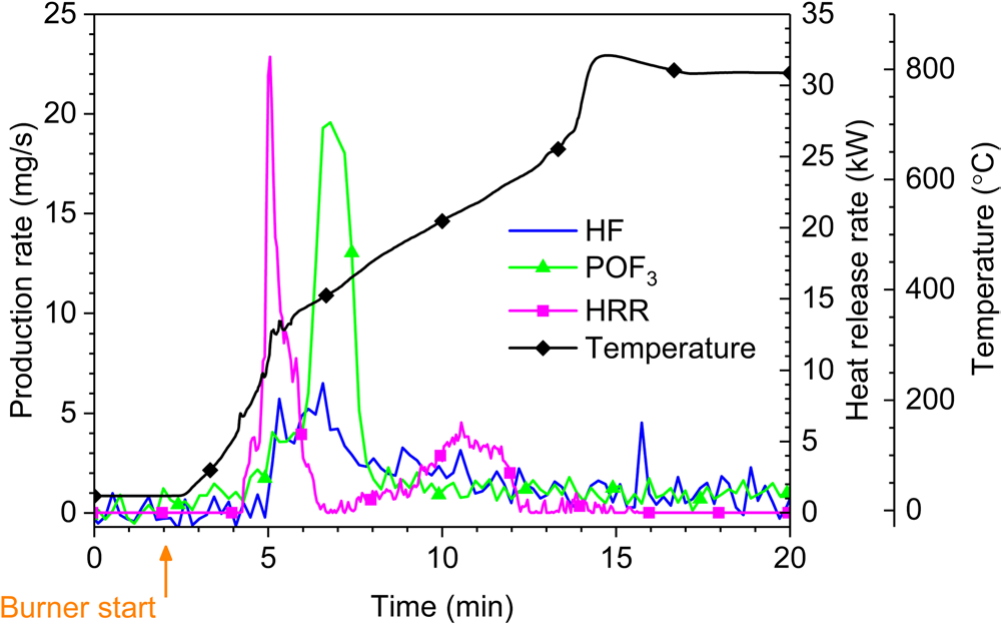
Results for a test with 5 type A cells at 0% SOC showing HF and POF_3_, HRR and average surface temperature of the battery cells.

In Fig. 3 the HRR, the average surface temperature of the five cells as well as the HF and POF_3_ production rates are shown for type A cells at 0% SOC. The POF_3_ curve is less noisy than the HF curve due to different signal-to-noise ratios of the FTIR instrumentation at the different wavenumbers. There is a secondary peak in HRR approximately 5 minutes after the main heat event, this peak does not correspond to any peaks in the mass flow of HF or POF_3_. The explanation for this could be that the second peak in the heat release rate involves burning of mainly non-fluorine containing compounds. The temperature curve shows a rapid increase above the melting temperature of the alumina cell case at about 660 °C. At these temperatures the alumina is molten and has formed a puddle on the burner bed beneath the battery cells. The thermal conditions in and around the thermocouples and the remains of the batteries have therefore changed considerably causing the apparent temperature increase.

**Figure 4:**
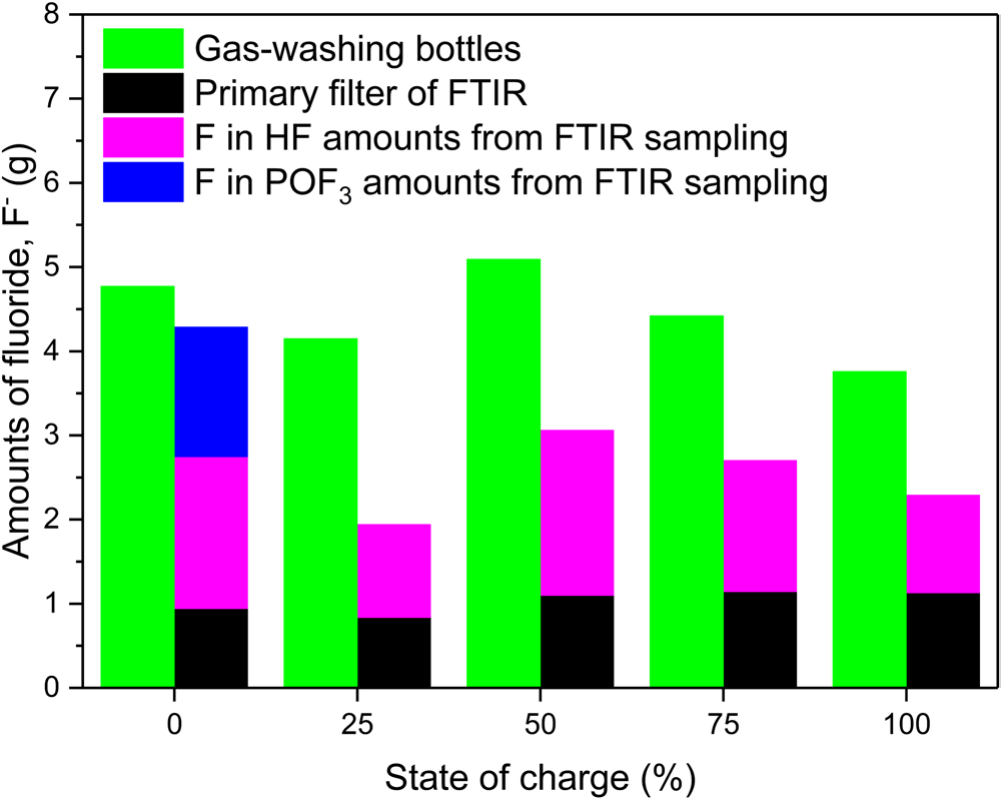
Total amount of measured fluoride, F^-^, for type A, for 0–100% SOC with intermediate steps of 25%. The amount of F^-^ from the FTIR is calculated from the measurement results for POF_3_ and HF, while the amount of fluoride from gas-washing bottles and primary filter analyses is measured as water soluble fluoride.

In addition to the time resolved measurements with the FTIR, gas-washing bottles were used to determine the total fluorine content in the gas emissions during the tests. A comparison between the different measurement methods used can be seen in Fig. 4 for type A cells. Note that the FTIR measurements are performed only to detect HF and POF_3_, other fluoride compounds are not included. It is interesting to note that for 0% SOC the total amount of fluoride measured by the gas-washing bottle technique matches rather well with the FTIR and primary filter analysis. For other SOC values the fluoride content is higher from the gas-washing bottle measurements. Still, the general trend observed in the FTIR measurements for different SOC values is more or less confirmed by the gas-washing bottle measurements.

Gas-washing bottles were also used for some of the tests involving battery types B and C. These batteries showed higher amounts of released HF compared to type A. The ratio between the total values of released flouride from FTIR plus filter analysis and from the gas-washing bottles for type B and C was between 0.89 and 1.02, indicating a better correlation between FTIR and gas-washing bottles measurement when HF gas emissions are higher.

The total amount of POF_3_ measured by FTIR for type A at 0% SOC was 2.8 g (for 5-cells) and 3.9 g (for 10 cells). Hence, the normalized total POF_3_ production was 15–22 mg/Wh of nominal battery energy capacity. Abuse studies measuring POF_3_ are few, Andersson *et al*.^46^ found both HF and POF_3_ when burning mixtures of propane and Li-ion battery electrolytes with a HF:POF_3_ production ratio between 8:1 and 53:1. Besides HF and POF_3_ measurements, several distinct non-assigned peaks were found in the FTIR measurements, e.g. at 1027 cm^−1^ and 1034 cm^−1^, which have also been seen in other studies^46^. They are compatible with the typical C-O stretching energies of low molecular weight alcohols in gas phase but also with in-plane stretching of aromatic compounds. This indicates the complexity and the limited knowledge in this area.

### Water mist measurements

In order to study the effects of water on gas emissions, fire tests have also been performed where a water mist was applied during the fire. The reason for this experiment is that water is the preferred extinguishing agent for a lithium-ion battery fire. The intention in this study was however not to extinguish the fire completely. One potential problem regarding the use of water mist is that the addition of water may, in principle, increase the rate of formation of HF, see Eqs (2) and (3).

**Figure 5:**
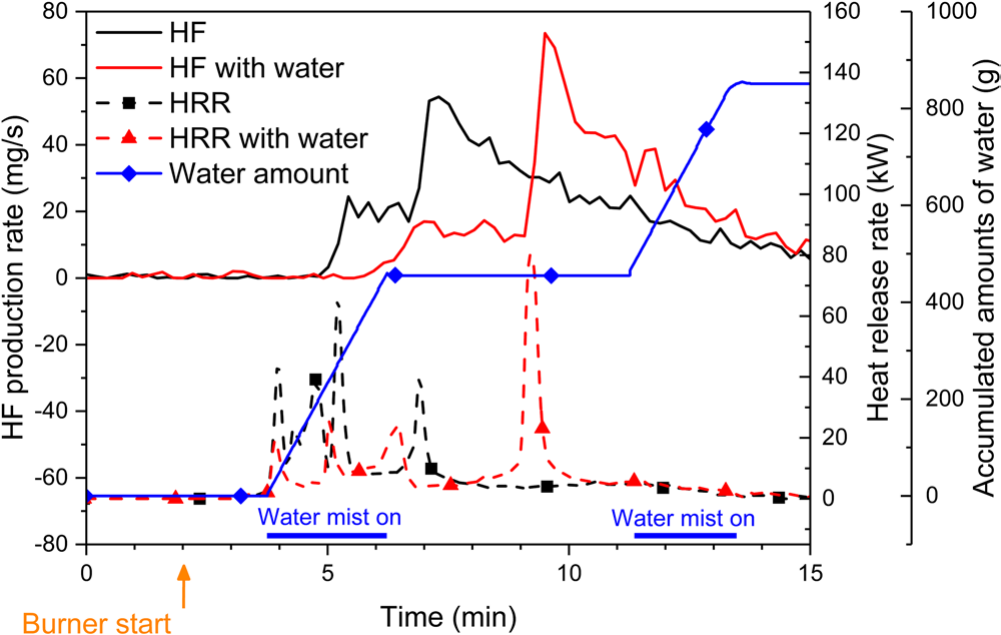
Results for type B cells at 100% SOC with and without the use of water mist.

Figure 5 shows the results for type B cells with and without exposure to water mist, note that both the HRR and HF production are delayed when water mist is used. In this limited study, the peak of the HF production rate increased by 35% when using water, however no significant change in the total amounts of the HF release could be seen. A similar result has been reported in a previous study^28^. The water mist was applied during two different periods of time, as marked in Fig. 5, adding a total of 851 g of water in the reaction zone, however, several other large sources of water were also present in the experiment, i.e. water production from the propane combustion and from humidity in the air. The water mist is cooling the fire and the top surface of the pouch cell was for some time partly covered with liquid water; this is the reason that the battery fire is delayed as seen in Fig. 5. The water mist might actually also clean the air by collecting fume particles and HF can be bound to water droplets, thus possibly lowering the amount of HF in the smoke duct and increasing the non-measured amount of very toxic hydrofluoric acid on the test area surfaces (e.g. walls, floor, smoke duct walls).

## Repeatability

Repeated tests were performed for battery types A-C for selected SOC-levels. Some of the repetitions included a variant, e.g. including water mist; see Methods. In Fig. 2 all available test data are presented. Since the test repetitions are not clearly observable in Fig. 2 the results are also presented in Table 3 showing the mean values and standard deviations and the number of performed tests. While the ranges in Table 2 include data for all tested SOC-values, Table 3 shows test data for repeated measurements including repetition variants.

**Table 2:** Main test results normalized to nominal energy capacity, when applicable including various SOC-levels.

Battery	Nominal energy capacity (Wh)	Normalized total HF detected with FTIR (mg/Wh)	Normalized maximum HRR (W/Wh)	Normalized THR (kJ/Wh)
A	128	15–25	243–729	17–19
B	128	150–198	78–633	45–50
C	112	43–160	116–491	66–75
D	92	12–24	207–315	27–30
E	132	52	235	50
F	138	55	384	50
G	124	15	460	28

**Table 3:** Detailed results for all available repetitions. Values presented as mean values followed by the standard deviation, in case the data parameter was not measured in all tests the value in bracket declares the number of available tests used for the specific data parameter value.

Battery	SOC (%)	Number of tests	Normalized total HF detected (mg/Wh)		Normalized maximum HRR (W/Wh)	Normalized THR (kJ/Wh)
			From FTIR	From gas-washing bottles		
A	100	6	19.8 ± 1.2 [3]	29.1 ± 3.1 [5]	612 ± 102	18.1 ± 0.46
	50	7	18.5 ± 3.9 [6]	36.7 ± 3.3 [6]	416 ± 39 [6]	18.0 ± 0.61 [6]
	0	2	21.6 ± 1.5	38.3 ± 1.6	214 ± 53	16.8 ± 0.66
B	100	4	166.8 ± 11.5	191.3 ± 11.3 [2]	538 ± 77	46.9 ± 1.9
C	100	3	53.9 ± 2.0 [2]*	N/A	461 ± 27	69.5 ± 2.6
	50	3	141.3 ± 26.3 [2]*	N/A	149 ± 5	70.5 ± 4.9

^*^For FTIR data for battery type C, one data point of 50% and one data point at 100% SOC are excluded as outliers since they were low due to that a pre HF-saturation was not applied in the test, see Methods.

**Figure 6:**
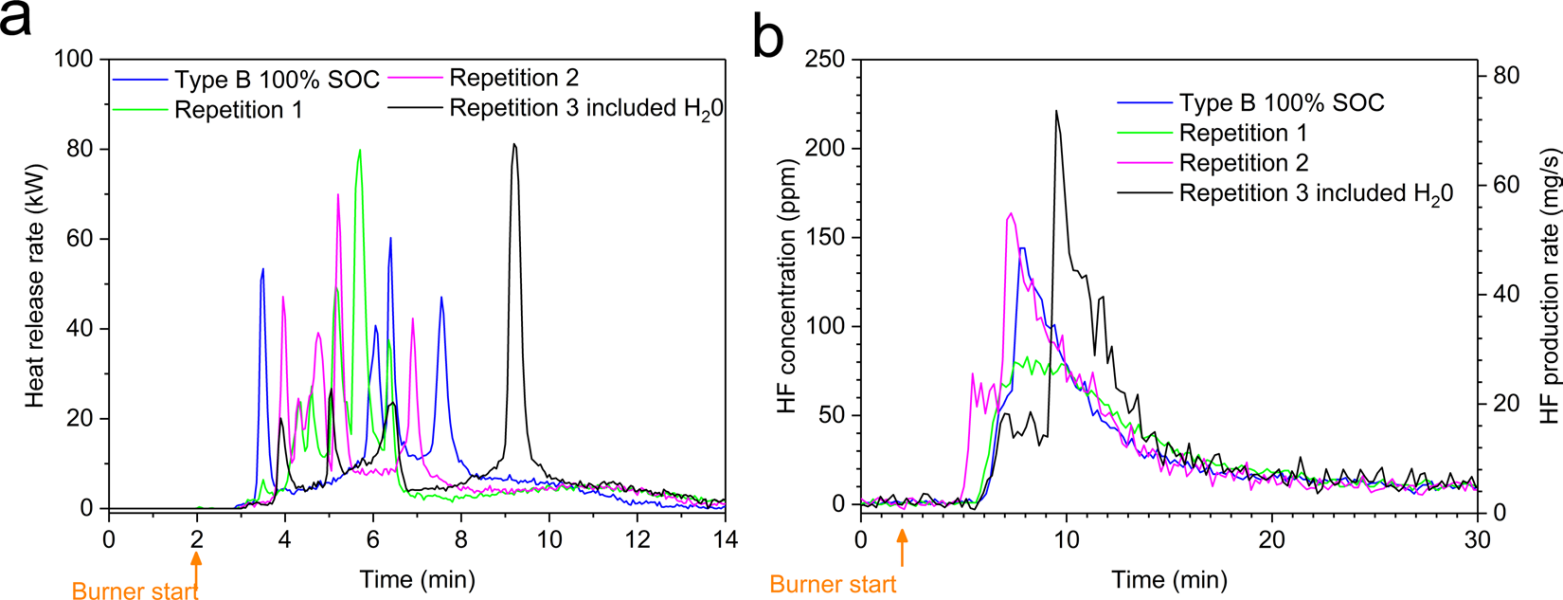
Repeatability for four tests of type B cells at 100% SOC, (**a**) shows the heat release rate (burner HRR contribution is subtracted) and (**b**) shows the HF release, both as the measured concentrations as well as the calculated HF production rates.

Figure 6 shows the repeatability results for four tests of battery type B for 100% SOC. The time evolution of HRR varies in the fire tests as seen in Fig. 6a. In fire tests there are always natural variations, however comparing the tests with 100% SOC, in Fig. 6a, with those with lower SOC-values presented in Fig. 1a, the repeatability of the 100% SOC tests is significant. The third repetition (black line) in Fig. 6a is delayed due to that it included an application of water mist, as discussed above. Although the appearance of the HRR plots of the four tests differs in Fig. 6a the THR (the integrated HRR) values are rather similar. Fig. 6b shows the HF release for the same four tests of type B at 100% SOC. Repetition 2 and 3 were performed in the third test period, without secondary FTIR filter, and therefore Repetition 2 occurs earlier while Repetition 3 is delayed due to the applied water mist, as discussed above. For the four tests of type B at 100% SOC the mean value of the total FTIR detected HF release is 166.8 mg/Wh with a standard deviation of 11.5 mg/Wh, as seen in Table 3. Comparing Fig. 1b and Fig. 6b, shows that for 100% SOC the HF release is faster and reaches a higher value. Repetition 1 in Fig. 6b shows lower HF release peak values, however, the total HF release value from the FTIR measurement of 168 mg/Wh is close to the average value (166.8 mg/Wh, as seen in Table 3).

## Conclusions

This study covered a broad range of commercial Li-ion battery cells with different chemistry, cell design and size and included large-sized automotive-classed cells, undergoing fire tests. The method was successful in evaluating fluoride gas emissions for a large variety of battery types and for various test setups.

Significant amounts of HF, ranging between 20 and 200 mg/Wh of nominal battery energy capacity, were detected from the burning Li-ion batteries. The measured HF levels, verified using two independent measurement methods, indicate that HF can pose a serious toxic threat, especially for large Li-ion batteries and in confined environments. The amounts of HF released from burning Li-ion batteries are presented as mg/Wh. If extrapolated for large battery packs the amounts would be 2–20 kg for a 100 kWh battery system, e.g. an electric vehicle and 20–200 kg for a 1000 kWh battery system, e.g. a small stationary energy storage. The immediate dangerous to life or health (IDLH) level for HF is 0.025 g/m^3^ (30 ppm)^22^ and the lethal 10 minutes HF toxicity value (AEGL-3) is 0.0139 g/m^3^ (170 ppm)^23^. The release of hydrogen fluoride from a Li-ion battery fire can therefore be a severe risk and an even greater risk in confined or semi-confined spaces.

This is the first paper to report measurements of POF_3_, 15–22 mg/Wh, from commercial Li-ion battery cells undergoing abuse. However, we could only detect POF_3_ for one of the battery types and only at 0% SOC, showing the complexity of the parameters influencing the gas emission. No POF_3_ could be detected in any of the other tests.

Using water mist resulted in a temporarily increased production rate of HF but the application of water mist had no significant effect on the total amount of released HF.

The research area of Li-ion battery toxic gas emissions needs considerable more attention. Results as those presented here are crucial to be able to conduct a risk assessment that takes toxic HF gas into account. The results also enable strategies to be investigated for counteractions and safety handling, in order to achieve a high safety level for Li-ion battery applications. Today we have a rapid technology and market introduction of large Li-ion batteries but the risks associated with gas emissions have this far not been possible to take into consideration due to the lack of data.

## Methods

**Figure 7:**
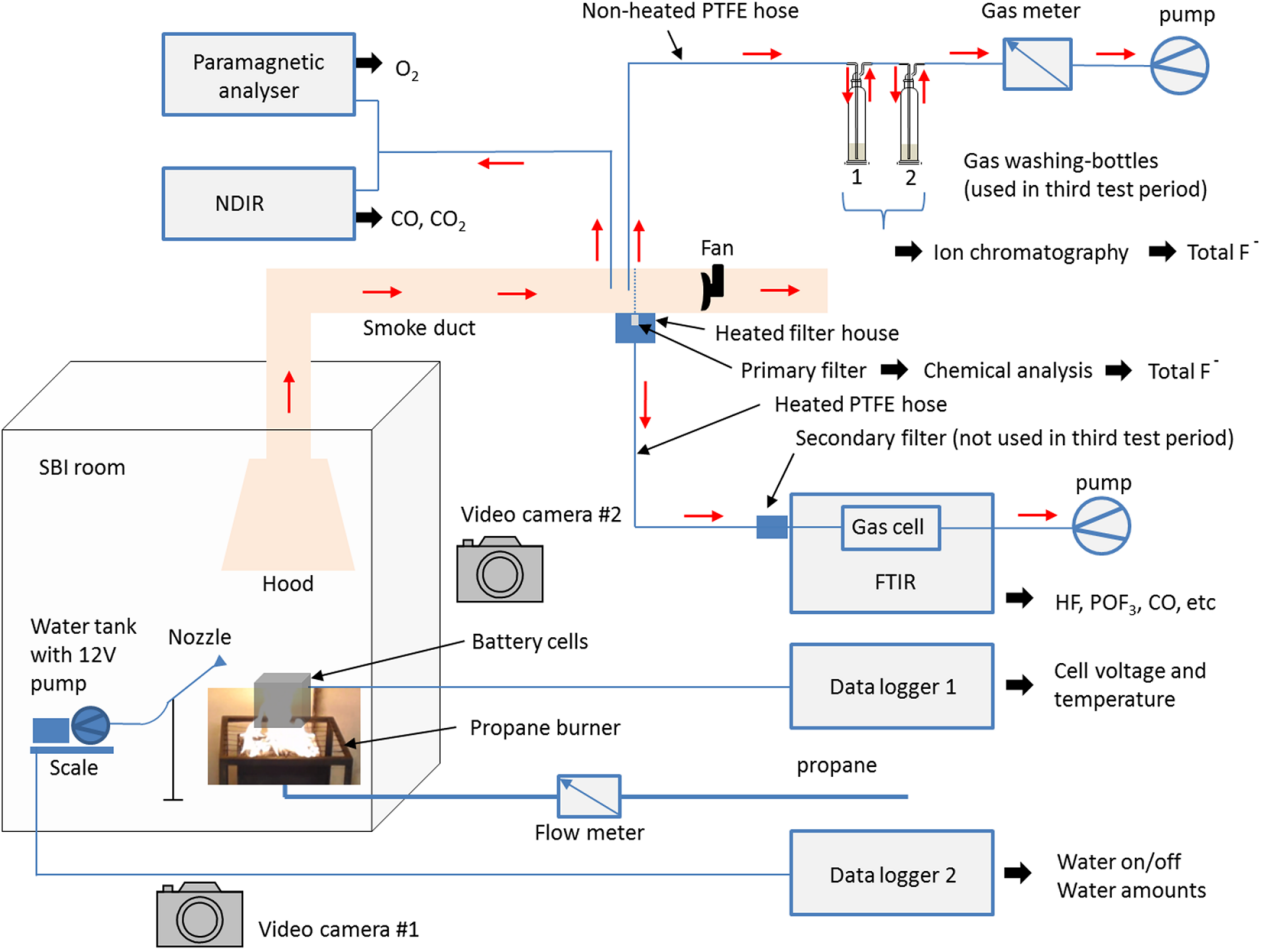
Schematic illustration of the experimental setup.

Seven types of Li-ion batteries were exposed to an external propane fire. Fire characteristics, gas emissions, battery temperatures and cell voltages were measured. In total 39 fire tests were conducted of which 20 were within the base test matrix, 19 were repeated measurements of selected battery types and SOC-levels of which 10 included a variant, e.g. water mist for fire-fighting. The amounts of emitted fluoride gases were measured with two parallel and independent techniques, FTIR (time resolved concentration measurements and total values achieved by integration of the time resolved curve) and gas-washing bottles (total values). The experimental setup is schematically shown in Fig. 7. The gas collecting system and measurement system of the *Single Burning Item (SBI) method* (EN 13823^54^), which is normally used for reaction-to-fire classification of construction products according to EN 13501-1^55^ was used in the tests. The tests were performed in three different test periods; the second test period was conducted about 1 year after the first and the third test period was conducted about 2.5 years after the first. Each test period involved several days of testing. The measurement equipment, as specified in the text below, was somewhat varying between the three test periods.

### Batteries

Six different types of Li-ion battery cells, type A-F, and one Li-ion battery pack, type G, were tested as seen in Table 1. The number of cells used in each test was varied in order to achieve similar electrical energy capacity per test. The batteries were placed on wire gratings just above a 16 kW propane burner. The wire grating was made of steel wire about 2 mm thick over a surface of about 300 × 300 mm. The quadrants of the grating were 40 × 100 mm. The cells were not electrically connected to each other (except the laptop packs of type G, see note in Table 1). Type A-F was pure battery cells while type G was a complete laptop battery pack which included plastics box, electronics and cables. The chemical content of the polymer materials in the auxiliary components of the battery pack of battery type G is not known. It is possible, however not likely, that fluorine was included in some of the components, which in that case could have resulted in the production of HF. For battery type A, 5 cells/test was used except in two variant tests in which 10 cells/test were used.

The influence of different state of charge was investigated, for some battery types the complete SOC-window ranging from 0% to 100%, with intermediate steps of 25%, was investigated. The SOC levels included for each battery type and the numbers of repetitions per test type, i.e. the fire test matrix, is seen in Table 4. All parameters were not measured in all of the tests. Measurement of HRR and corresponding THR was conducted in 38 tests, FTIR in 35 tests and gas-washing bottles were used in 19 tests.

**Table 4:** Detailed test matrix of the fire tests.

Battery	Number of tests per SOC-level					Number of tests
	0%	25%	50%	75%	100%	
A	1 + 1*	1	3 + 4*	1	3 + 3*	17
B	1	1	1	1	3 + 1*	8
C	1	1	3	1	2 + 1*	9
D			1		1	2
E					1	1
F					1	1
G					1	1
Total number of tests						39

^*^repetition includes a variant, e.g. water mist or 2 × 5-cell-pack (for battery type A).

The selected SOC level in each test was set using a charge/discharge procedure using ordinary laboratory equipment as well as dedicated battery test equipment, i.e. a *Digatron battery tester* and *Metrohm Autolab PGSTAT302N* with 20 A booster module. The cells were first fully charged by constant current followed by constant voltage (CC-CV) according to the manufacturer’s instructions. For cells intended for tests with less than 100% SOC, the cell was discharged to the selected SOC level, using constant discharge current (CC). A relative low current rate, about C/5, was used and voltage and current rates were within the manufacturer limits. In most cases each battery type was tested during the same test period. However, the tests for type C and D were split in several test periods, for type C repetitions on 50% SOC were conducted in all three test periods, and for type B repetitions at 100% SOC were made in two test periods, the latter one included a water mist test.

**Figure 8:**
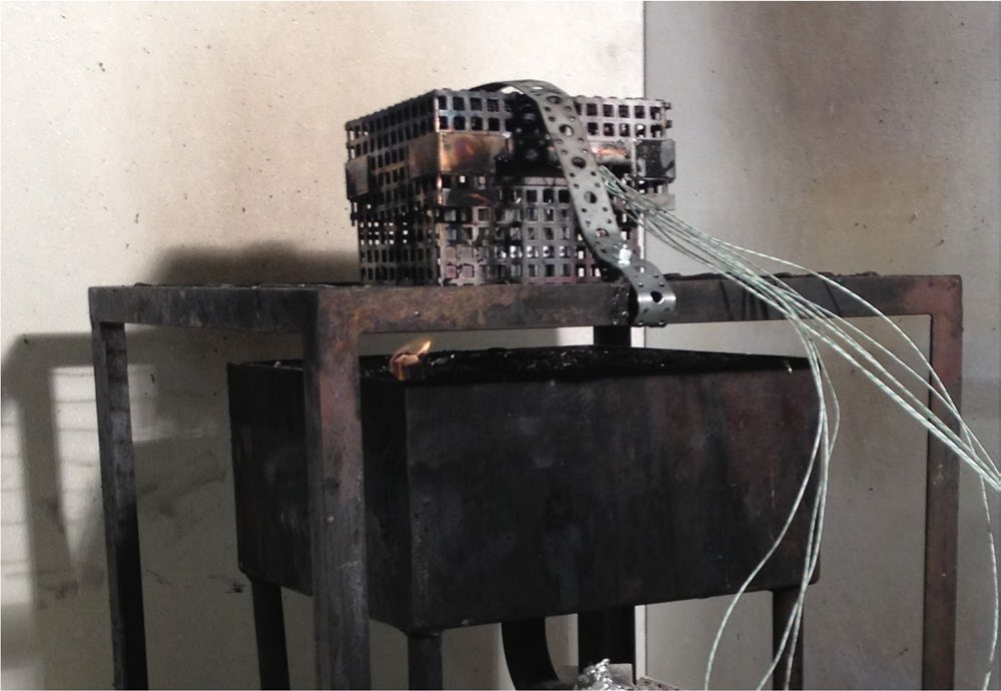
Photo of test type A, showing the 5-cell-pack inside a steel-net-box placed on the wire gratings. The sand bed for the propane burner is underneath the wire grating, a pilot flame (seen in front left corner of the burner) is used to ignite the propane gas.

All batteries were unused and the calendar life time of the cells before the tests were approximately 6–12 months for type A, F and G and between approximately 2–3 years for type B-E. The pouch cells; type B, C and F was mechanically tied together with steel wires (0.8 mm diameter). The type A hard prismatic cells were tight together in packs of five cells, “5-cell-pack”, using steel straps (1 × 13 mm). The hard prismatic and cylindrical cells were placed in boxes to protect test personnel from potential projectile hazards in case of cell explosions due to excessive pressure. The 5-cell-pack of type A was placed standing up, with the cell safety vents releasing straight upright in direction to the hood and smoke duct, inside a custom-made steel-net-box, see Fig. 8. Additionally, the 5-cell-pack of type A was fastened to the bottom of the steel-net-box with steel wire (0.8 mm diameter) in the corners to avoid it moving around due to e.g. explosion/rupture/venting. Type D and E cells were placed standing up in custom-made boxes made of non-combustible silica board and steel net at the top and bottom. Type G was placed in a steel net. The protective boxes and steel net were fastened in the wire gratings with steel wire and steel straps to avoid movement due to response to the fire. Care was taken to avoid external short circuiting when placing the battery on the wire gratings as well as avoiding accidental external electrical inter-cell-connections, e.g. for pouch cells the electrical tab terminals were cut. Still the battery test setup allowed that the separators and electrical insulation in the cells could melt due to the heat exposure which could cause various internal and external electrical contacts.

The battery surface temperature was measured with several type K thermocouples; the number of sensors varied for the different battery types. Battery cell surface temperature values presented in this paper are average values over the cell. Cell voltages were measured for type A, B, C and F battery tests. Cell voltage and thermocouple readings was sampled with 1 Hz using two types of data loggers, *Agilent 34972 A using an Agilent 34902 A reed multiplexer module* (for the third test period) and *Pico Technology ADC-24* (for the first and second test period).

### Test procedure

The propane burner was started 2 minutes into each test, as indicated with arrows in the result figures in the paper. The burner was active as long as there was a heat contribution from the burning batteries; therefore, the burner was active for different durations of time for different batteries and SOC-levels. When the heat release from the batteries was no longer detectable, the power of the propane burner was doubled, i.e. to 32 kW, in order to be sure to fully burn out any residues of the batteries, for increased personnel safety. The fire emissions were collected in the hood and transferred in the smoke duct having a ventilation flow of 0.4 m^3^/s, with the exception that 0.6 m^3^/s was used in two tests with 100% SOC for type C. For these cases the values were scaled down to the lower flow values making the results from the two flow rates comparable. The SBI-room, see Fig. 7, had a ventilation inlet from an adjacent indoor laboratory hall (which had fresh air inlet from the ventilation system in the building), supplying ambient air with temperature about 20 °C entering beneath the propane burner. We consider the amount of ambient air to be sufficient to provide an oxygen-rich environment and thereby consider the battery fire as well-ventilated. However for some tests, during the rapid and energetic gas outbursts, a full combustion might not have occurred in these short time periods.

All tests were video recorded and for the majority of the tests an additional camera was used set at 90 degree angle from the other video camera, allowing simultaneous recording from two sides of the battery fire.

A part of the smoke duct flow was sampled to a *Servomex 4100 Gas purity analyser* where the oxygen content was measured by a *paramagnetic analyser* and CO and CO_2_ were measured by a *non-dispersive infrared sensor (NDIR)*. By combing these two measurements, the heat release rate (HRR) is calculated using the oxygen consumption method corrected by CO_2_^54^. Each test day started with a blank test, i.e. using only the propane burner, to measure the HRR of the burner alone and measure blanks for FTIR and gas-washing bottles. In the presented HRR values of the battery tests the burner contribution to the HRR (about 16 kW, with slight daily variations, established by the blank tests) has been subtracted. The combined expanded uncertainty is ±5 kW for the HRR-values. By integrating the HRR values over the entire test, subtracting the HRR from the burner, the total heat release (THR) from the battery cells could be established. The oxygen consumption method is common in fire calorimetry, however when using it with batteries, the joule heating from electrical discharge within the cells is not accounted for, therefore the values of HRR and THR do not include the Joule heating. During the external fire tests, it is difficult to measure how much a battery cell is electrically discharged when the separator is melting. The energy ratios presented in Fig. 2b do not include any Joule heating as clearly stated by its definition. For 0% SOC the influence from Joule heating is in principle zero, however small amounts of joule heating might possibly be liberated when going to zero voltage even though other processes might occur. Li-ion cells can also release oxygen during thermal runaway and this could affect the measured O_2_ levels. The amount of oxygen release varies for different electrode materials, e.g. LFP typically releases less oxygen than LCO. However, the ventilation flow is large and the O_2_ released from the battery cells is regarded as negligible.

### Gas measurements

Besides the gas measurements in the SBI apparatus, measurements of gases were also conducted by online Fourier transform infrared spectroscopy (FTIR). The FTIR offers broad and diverse spectra of gases, the focus was however on fluoride gas emissions. The FTIR used was a *Thermo Scientific Antaris IGS analyzer (Nicolet)* with a gas cell. The gas cell was heated to 180 °C and had a volume of 0.2 L, 2.0 m path length and a cell pressure of 86.7 kPa which was maintained during the tests. The spectral resolution of the FTIR was 0.5 cm^−1^ (accuracy 0.01 cm^−1^) and 10 scans where used to collect a spectrum every 12 s, giving both accurate intensity, as well as relatively rapid measurements with its five spectrum per minute rate. A part of the duct flow, taken along the full duct pipe width (in the mid height of the pipe) from around 15 sampling holes (about 2 mm diameter, directed opposite to flow, pipe end was closed), was taken to online FTIR measurement. This sub-flow was extracted through a primary filter inside a heated filter house (180 °C) and then extracted through an 8.5 m sampling PTFE hose, heated to 180 °C, and then through a secondary filter and finally through the gas cell of the FTIR. The sub-flow was selected to be 3.5 L/min using a pump located after the FTIR gas cell. Between each test the FTIR sampling system was flushed with N_2_ gas and a new background spectrum was measured. There is a natural delay time between the FTIR and the heat release measurement. In order to time synchronize them the (CO_2_ measurements from both the FTIR and the NDIR) part of the heat release rate measurement, were overlayed.

One primary filter (M&C ceramic filter, type “F-2K”) was used per test and was chemically analysed for fluoride content after the test. It is known that HF may be partly adsorbed by this type of filter^56^. The fluoride amount absorbed by the filter was determined by leaching the filter in an ultrasonic water bath for at least 10 min and thereafter the fluoride content in the water was measured by ion chromatography with a conductive detector, according to the method B.1 (b) of the SS-ISO 19702:2006 Annex B standard. The amount of HF is calculated by assuming that all fluoride ions present in the filter derives from HF. The secondary filter (M&C sintered steel filter), heated to 180 °C, was the same in all tests in the first and second test period. In the third test period the secondary filter was removed in order to decrease delay time and losses. The third test period started with burning 10 cells of type A in order to saturate the FTIR sampling system with HF and it was conducted because in the first and the second test period the first tests had indicated low HF values, HF was potentially lost during saturation of the gas collecting system.

The FTIR was calibrated^29,57^ for HF and POF_3_. The minimum detection limit (MDL) for HF was 1.7 ppm and the limit of quantification (LOQ) was established to 5.7 ppm. The detection limit for POF_3_ was 6 ppm^29^. PF_5_ was also qualitatively detectable by the FTIR^29^ but not quantitatively calibrated. A classical least square (CLS) method was used for the quantification of HF and POF_3_ using the spectral bands specified in Table 5. The relative error of the HF prediction is lower than 10 rel-%.

**Table 5:** FTIR spectral band used for measurements of POF_3_ and HF.

Spectral bands (cm^−1^)	Type of band
POF_3_	
868–874	P-F symmetric stretching mode^20^
1413–1418	P-O stretching mode^20^
HF	
4172–4175	HF R-branch stretching mode^58^
4202–4203	HF R-branch stretching mode^58^

For all measurements, except type G, the measured ppm levels of HF were above the detection level. For POF_3_, the maximum concentration was 11 ppm (5-cells) and 19 ppm (10-cells).

When the FTIR measurement stopped, HF levels were, in some of the tests, still somewhat above the detection limit, even though no HRR contribution was measured from the batteries. It is also possible that the HF was temporarily clogged in the sampling system. Some HF might not have been collected in the measurements and the effect of this error is largest for the batteries that give the lowest values. Thus the reported values might underestimate the released gas emissions.

In order to further improve the accuracy of the FTIR measurements, a data offset determination and a subsequent adjustment of the HF values was performed. The improvement was greatest for tests with lower concentrations, closer to the MDL value, e.g. type A with 5 cells with low values during relatively short periods of time. With 10 cells per test, the type A batteries gave higher signal-to-noise levels. The FTIR measurements started around 8 minutes before the burner was started. The calculated average HF ppm noise level was treated as an offset that had both negative and positive values, ranging from extreme values of about −2 to 3.5 ppm. This offset was compensated for by assuming a constant offset value and adding positive or negative offset values to the total HF release value. Note that the reported concentration values in ppm are only valid for the measurements in the smoke duct of our specific test equipment and method. The HF and POF_3_ concentration values (in ppm) were used for calculating the corresponding production rates (in mg/s) using the ideal gas law and taking into account the measured ventilation flow rate in the smoke duct.

In the third test period the total amounts of water soluble fluorides were determined using gas-washing bottle technique. This was made in order to validate the results from the FTIR measurements with a separate measurement technique. The water soluble fluorides were collected in the bottles and the amount of HF was calculated by assuming that all fluoride ions present derives from HF. The sample gas was extracted from the center of the smoke duct using a non-heated 6 mm (o.d.) diameter PTFE sampling tube with a length of about 1.5 m. The sampling was made using two gas-washing bottles connected in series each containing 40 mL of an alkaline buffer solution (20 mM Na_2_CO_3_/20 mM NaHCO_3_). The second bottle was used to capture any losses from the first bottle. The sampling flow was 1.0 normal-L/min and the total sampled volume during a test was measured by a calibrated gas volume meter. The sampling flow rate was checked before the start of each test using a *Gilian Gilibrator-2 NIOSH Primary Standard Air Flow Calibrator* gas flow meter. The procedure during a test was to continuously sample during the full test time. When the test was completed, the sampling tube was disconnected from the exhaust duct to allow rinsing of the tube with buffer solution, about 30 mL in the first gas-washing bottle, to collect any fluoride deposited on the inner walls of the tubing, in order to minimize losses in the tube. Since the tube was rinsed, heating of the tube was not necessary (any condensation in tube was collected anyhow). Analysis of fluorine content of the absorption solutions was made using High Performance Ion Chromatography (HPIC). The contents of the two gas-washing bottles were analyzed separately. The bottles were rinsed with distilled water between each test in order to minimize any interference between tests.

### Water mist test

In the water mist tests, a custom-made equipment was constructed, including a 12 V automotive pump and water container which was placed on a scale measuring the weight of the water. The scale readings and the on/off manual switching (of the 12 V) was recorded with 1 Hz using *Pico Technology ADC-24* with a custom-made *LabVIEW* program. The water mist was sprayed on or above the batteries using a metal nozzle. In order for precise time synchronization, the on/off 12 V signal was recorded by both data loggers (data logger 1 and data logger 2). A blank test, i.e. using only the propane burner and without batteries, was performed in order to calibrate the setup. The water flow was around 190 g water per min and consisted of deionized water.
