# Author Correction: Toxic fluoride gas emissions from lithium-ion battery fires

**DOI:** 10.1038/s41598-018-22957-8

**Published:** 2018-03-22

**Authors:** Fredrik Larsson, Petra Andersson, Per Blomqvist, Bengt-Erik Mellander

**Affiliations:** 1 Department of Physics, Chalmers University of Technology, Kemivagen 9, SE-41296 Gothenburg, Sweden; 2 Safety and Transport, RISE Research Institutes of Sweden, Brinellgatan 4, SE-50115 Boras, Sweden

Correction to: *Scientific Reports* 10.1038/s41598-017-09784-z, published online 30 August 2017

This article contains a typographical error in the Conclusions section, where:

“The immediate dangerous to life or health (IDLH) level for HF is 0.025 g/m^3^ (30 ppm)^22^ and the lethal 10 minutes HF toxicity value (AEGL-3) is 0.0139 g/m^3^ (170 ppm)^23^.”

should read:

“The immediate dangerous to life or health (IDLH) level for HF is 0.025 g/m^3^ (30 ppm)^22^ and the lethal 10 minutes HF toxicity value (AEGL-3) is 0.139 g/m^3^ (170 ppm)^23^.”

